# The socio-economic drivers of bushmeat consumption during the West African Ebola crisis

**DOI:** 10.1371/journal.pntd.0005450

**Published:** 2017-03-10

**Authors:** Isabel Ordaz-Németh, Mimi Arandjelovic, Lukas Boesch, Tsegaye Gatiso, Trokon Grimes, Hjalmar S. Kuehl, Menladi Lormie, Colleen Stephens, Clement Tweh, Jessica Junker

**Affiliations:** 1 Department of Primatology, Max Planck Institute for Evolutionary Anthropology, Leipzig, Germany; 2 Institute for Sociology, University of Leipzig, Leipzig, Germany; 3 German Centre for Integrative Biodiversity Research, Leipzig, Germany; 4 Forestry Development Authority, Wheintown, Mount Barclay, Liberia; 5 School of Biological and Physical Sciences, University of Nairobi, Nairobi, Kenya; Common Heritage Foundation, NIGERIA

## Abstract

Bushmeat represents an important source of animal protein for humans in tropical Africa. Unsustainable bushmeat hunting is a major threat to wildlife and its consumption is associated with an increased risk of acquiring zoonotic diseases, such as Ebola virus disease (EVD). During the recent EVD outbreak in West Africa, it is likely that human dietary behavior and local attitudes toward bushmeat consumption changed in response to the crisis, and that the rate of change depended on prevailing socio-economic conditions, including wealth and education. In this study, we therefore investigated the effects of income, education, and literacy on changes in bushmeat consumption during the crisis, as well as complementary changes in daily meal frequency, food diversity and bushmeat preference. More specifically, we tested whether wealthier households with more educated household heads decreased their consumption of bushmeat during the EVD crisis, and whether their daily meal frequency and food diversity remained constant. We used Generalized Linear Mixed Models to analyze interview data from two nationwide household surveys across Liberia. We found an overall decrease in bushmeat consumption during the crisis across all income levels. However, the rate of bushmeat consumption in high-income households decreased less than in low-income households. Daily meal frequency decreased during the crisis, and the diversity of food items and preferences for bushmeat species remained constant. Our multidisciplinary approach to study the impact of EVD can be applied to assess how other disasters affect social-ecological systems and improve our understanding and the management of future crises.

## Author summary

The consumption of wild animal meat, commonly known as bushmeat, is widespread throughout tropical regions. Bushmeat provides an essential source of protein and income for human livelihoods. However, its consumption is linked to the transmission of zoonotic diseases, such as Ebola, and its over-harvest is a major threat to many wildlife species. The bushmeat trade therefore encompasses a broad range of socio-economic and ecological issues. As such, we think it is highly important to use an interdisciplinary approach to investigate the drivers of bushmeat consumption, to improve our understanding and management of future crises. Our analysis of household interview data collected during two surveys across Liberia shows that there was an overall decrease in bushmeat consumption during the recent Ebola crisis. However, the consumption of bushmeat in wealthier households decreased less than in poorer households. In addition, we found that daily meal frequency decreased during the crisis, and the diversity of food items and preferences for bushmeat species remained constant.

## Introduction

The recent Ebola virus disease (EVD, [1]) epidemic that emerged in March 2014 in West Africa [2, 3] was the largest recorded, resulting in over 28,600 cases and 11,300 deaths in Guinea, Liberia and Sierra Leone [4]. EVD is a deadly zoonotic disease that is transmitted to humans through contact with the blood and other bodily fluids of infected wildlife, such as fruit bats, forest antelopes, and nonhuman primates [5, 6]. Although there are several contributing factors that caused the outbreak to rapidly expand, such as poverty [7, 8, 9] and weak medical infrastructure [8, 10], the harvest and butchering of bushmeat (i.e., wild animal meat) have been suspected as potential sources for initial spillover events in this [2, 11] and other EVD epidemics [12, 13].

Despite its health risks, bushmeat consumption is widespread throughout tropical regions and common in both rural and urban areas [14, 15], although the reasons for its consumption tend to vary between and within areas. In remote, impoverished, rural areas, bushmeat is often an essential source of animal protein that may contribute to food security, particularly where livestock and fish are inaccessible [16, 17, 18] or unaffordable [19]. In contrast, urban consumers are likely to choose bushmeat from a number of interchangeable animal protein sources, and for a variety of reasons, such as its low cost, preference of taste, or perception of prestige [16, 20]. Bushmeat also provides an important source of cash income for rural and forest dwellers who may depend on wildlife to alleviate periods of economic hardship (e.g. crop failures), or supplement their primary source of income, which is often agriculture [21, 22].

Along with the rapid growth of human populations, the extraction of wildlife for subsistence and commercial use has become a major biodiversity threat [23]. Since the 1970s, the abundance of large mammals in African protected areas has halved, largely due to over-hunting [24]. To make more profit, hunters prefer large animals, such as large antelopes, elephants, and great apes, whose low intrinsic rates of population growth make them extremely vulnerable to intensive hunting. Regardless of their scarcity, they are continued to be targeted and have already been hunted to the point of extirpation in a number of places [20, 25]. These unsustainable hunting practices ultimately lead to the defaunation of otherwise undisturbed forests and create “empty forests” [26]. The ecological impacts of over-hunting include the direct effect on the hunted populations (e.g. Tweh et al. [27] have documented this across Liberia), as well as indirect effects on ecosystem function and structure, which are more difficult to measure [20, 28]. For instance, Effiom et al. [29] found that the seedling layer in forest sites with widespread bushmeat hunting was significantly different from that of protected sites, suggesting that the loss of seed-dispersing primates in particular might impact forest regeneration processes and ultimately forest composition.

Indeed, the bushmeat trade encompasses a broad range of socio-economic and ecological issues, which highlights the need to use interdisciplinary approaches to better understand the links between the exploitation of natural resources and human socio-economic status [19, 30]. It is therefore imperative to identify the underlying drivers of bushmeat consumption to develop more effective, targeted conservation management strategies. Such strategies must aim to reduce the unsustainable harvest of bushmeat whilst improving human livelihoods and lowering the risk of zoonotic disease transmissions, such as EVD.

It is likely that due to social pressure and risk aversion, human behavior and local attitudes toward bushmeat consumption changed during the most recent and largest-ever recorded Ebola outbreak in West Africa [2]. Furthermore, the magnitude of these changes may have been influenced by different socio-economic factors. Here, we analyze household-level data on socio-economic status, wildlife consumption and eating habits, to answer the following research question: were wealthier, more educated or literate people more likely to change (1) bushmeat consumption, (2) number of meals per day, (3) food diversity, and (4) bushmeat preference during the Ebola crisis?

## Methods

### Study area

Our surveys were conducted across the entire country of Liberia. Following 14 years of civil conflict, the country’s economy had been growing rapidly in recent years [31]. However, Liberia is still among the poorest countries in the world (HDI rank 177/188, [32]), and was one of the countries hardest hit by the Ebola crisis [10, 31]. It is also found within the richest 5% of land area for threatened bird, amphibian and mammal species [33]. In addition, it is home to one of the most viable chimpanzee populations in West Africa, which is primarily threatened by hunting [27].

### Field data collection

We used two different data sources: (1) interview data collected in Liberia during a nationwide chimpanzee and large mammal survey from 2010 to 2012, and (2) interview data from a follow-up nationwide interview survey on socio-economic status and natural resource use of Liberian households during the Ebola crisis in 2015.

Between August 2010 and May 2012 a nationwide chimpanzee and large mammal survey was conducted on line transects that were systematically distributed across the country [27]. When travelling to these transects to record large mammal abundance, survey teams visited nearby villages to conduct interviews to collect data that served as the basis of this study (for more details see Tweh et al. [27]). In each location (i.e., village), one to ten household heads were interviewed. If the head of the household was absent at the time, the person otherwise responsible for the household was interviewed instead. A total of 275 household heads from 70 locations were interviewed during this survey.

**Fig 1:**
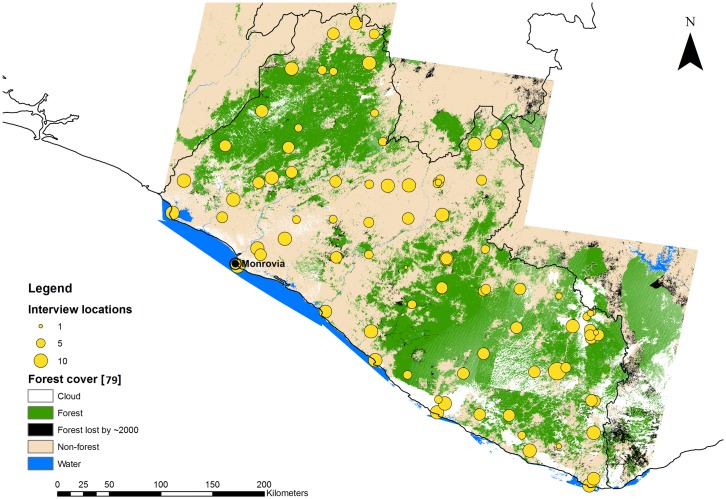
Map of Liberia showing the interview locations of the 2015 follow-up survey. The size of the circles depicts the number of households surveyed in each location. Forest cover based on Christie et al. [79].

A follow-up interview survey was conducted between January and June 2015 to gather information on the impact of Ebola on socio-economic status and natural resource use of Liberian households. The geographical distribution of interview locations during the follow-up survey in 2015 was based on the sampling locations from the 2010–2012 survey (Fig 1), but the respondents and households were not necessarily the same across surveys. The majority of the interview questions were paired with identical retrospective questions, which made it possible to collect information about the time period before the Ebola crisis. This was an important feature of the questionnaire because the 2010–2012 survey questionnaires were far less extensive (see S2 Appendix for the complete questionnaires used in the surveys). For this reason, data from the 2015 follow-up survey were mainly used in our data analysis. Overall, there was an overlap of 60 interview locations that were sampled during both household surveys.

### Analytical methods

#### Response variables

We ran Generalized Linear Mixed Models (GLMMs, [34]) for eight response variables to assess the changes in eating habits before vs. during the Ebola crisis. Specifically, we assessed the changes in (1) the number of meals eaten per day, (2) bushmeat consumption, (3) food diversity, and (4) bushmeat species preference (Table 1). In addition, we ran two Linear Mixed Models (LMMs, [34]) to investigate whether bushmeat and fish prices changed during the crisis, and one GLMM to investigate whether domestic meat prices changed. All but one response variable (number of meals per day) had to be processed before fitting them to the models; for more details see S1 Appendix.

**Table 1:** Description of the response variables used to analyze changes in eating habits and meat prices.

Models	Response variable	Measurement	Data source
Number of meals per day	Change in number of meals per day	Number of times a day respondents ate on average before and during the Ebola crisis	Follow-up interview survey (2015)
Bushmeat consumption	Change in frequency of bushmeat consumption	Frequency of bushmeat consumption before and during the crisis, ordered by ranks: (0) < once a month, (1) once a month, (2) once a week, (3) twice a week, (4) every second day, (5) every day, and (6) with every meal	Follow-up interview survey (2015)
	Change in proportion of people in the community that preferred to eat bushmeat	Proportion of people in the community that respondents thought preferred to eat bushmeat before and during the crisis, ordered by ranks: (0) very few people, (1) few people, (2) half, (3) > half, (4) everybody	
Food diversity	Change in number of food items consumed	Number of different food items consumed during a typical meal on a typical day before and during the crisis	Follow-up interview survey (2015)
	Change in number of food groups consumed	Number of different food groups (staples, vegetables, fruits, meat, fish and seafood, and oil) consumed during a typical meal on a typical day before and during the crisis	
Bushmeat preference	Change in preference for duiker meat	Likelihood of people preferring to eat duiker meat before and during the crisis	Nationwide chimpanzee and large mammal survey (2010–2012) and follow-up interview survey (2015)
	Change in preference for monkey meat	Likelihood of people preferring to eat monkey meat before and during the crisis	
	Change in preference for pangolin meat	Likelihood of people preferring to eat pangolin meat before and during the crisis	
Meat prices	Change in bushmeat prices	Bushmeat prices in Liberian Dollars (LRD) before and during the crisis^†^	Nationwide chimpanzee and large mammal survey (2010–2012) and follow-up interview survey (2015)
	Change in fish prices	Fish prices (LRD) before and during the crisis^†^	
	Change in meat prices	Domestic meat prices (LRD) before and during the crisis	

^†^Variable was log-transformed to normalize skewed distribution

#### Predictor variables

To test for an influence of our socio-economic factors on changes in eating habits, the following test predictors were included in our GLMMs: the interaction of time period (i.e., before vs. during the Ebola crisis) with: (1) monthly income, (2) number of years of education, and (3) literacy, as well as (4) time period alone (Table 2). The interaction was included for each test predictor because we hypothesized that the influence of the time period on our response variables would vary depending on each socio-economic factor. We included time period alone as a test predictor to confirm that changes in eating habits that could not be explained by the other test predictors were associated with the incidence of the EVD outbreak.

**Table 2:** Description of the test predictors and fixed-effects control predictor variables or their interactions. Their measurements, the type of data, data sources, the names of the models they were included in, the hypothesized effects on the response variables, and references to studies that have examined their effects before.

Variable name	Measurement	Type of data	Data source	Included in models for:	Brief explanation of hypothesized effect	References of studies that examined the effects before
Income*time period^†^^§^	Monthly income (LRD)	Continuous	Follow-up interview survey (2015)	Number of meals per day, bushmeat consumption, food diversity	Wealthier people are more likely to have a more diverse diet and higher protein intake.	[16, 37, 38, 39]
Education *time period^†^	Number of years of primary and secondary education	Continuous	Follow-up interview survey (2015)	Number of meals per day, bushmeat consumption, food diversity	Households with more educated household heads are more likely to be informed about EVD and therefore avoid bushmeat in their diet.	[19]
Literacy*time period^†^	Whether or not respondent is literate	Categorical	Follow-up interview survey (2015)	Number of meals per day, bushmeat consumption, food diversity	Households with literate household heads are more likely to be well informed about EVD and therefore avoid bushmeat in their diet.	[19]
Time period^†^	Periodization of two time blocks: before and during the Ebola crisis	Categorical	Follow-up interview survey (2015) and nationwide chimpanzee and large mammal survey (2010–2012)	Number of meals per day, bushmeat consumption, food diversity, bushmeat preference, meat prices	Changes in eating habits during the Ebola crisis are associated with the incidence of the Ebola outbreak.	This study
Ebola infections^‡^	Presence or absence of EVD-infected people in respondent’s community	Categorical	Follow-up interview survey (2015)	Number of meals per day, bushmeat consumption, food diversity	If Ebola is present in the community, respondents will likely be more fearful and therefore reduce or eliminate bushmeat from their diets as a precaution.	The authors are not aware of any studies that investigated this effect
Perceived risk of bushmeat consumption^‡^	Respondent’s opinion on whether or not EVD can be contracted from bushmeat	Categorical	Follow-up interview survey (2015)	Number of meals per day, bushmeat consumption, food diversity	Respondents will more likely eliminate bushmeat from their diets if they believe that they can contract Ebola from it.	[40, 41]
Perceived law enforcement^‡^	Respondent’s opinion on whether or not law enforcement effectively protects animals	Categorical	Follow-up interview survey (2015)	Number of meals per day, bushmeat consumption, food diversity	Respondents will more likely avoid consuming illegally hunted bushmeat if they think that law enforcement is effective.	[42]
Distance to roads^‡^	Euclidean distance from survey location to closest road	Continuous	Follow-up interview survey (2015)	Number of meals per day, bushmeat consumption, food diversity	Households in remote areas far from roads are more likely to rely on bushmeat as a protein source than households in areas with good road networks, where the latter have better access to bushmeat through markets, restaurants and other infrastructure. Proximity to roads in remote areas may also facilitate the access to forests for hunters.	[16, 43, 44, 45]
Distance to settlements^‡^	Euclidean distance from survey location to closest settlement.	Continuous	Follow-up interview survey (2015)	Number of meals per day, bushmeat consumption, food diversity	Households in remote, rural areas that are close to the forest are likely to have better access to bushmeat for their own consumption, whereas households that are far away from the forest are likely to obtain bushmeat from elsewhere.	[16, 43, 46]
Livestock consumption^‡^^§^	Amount of livestock owned and consumed by the household during 12 months prior to the interview (kg)	Continuous	Follow-up interview survey (2015)	Bushmeat consumption	Households that own more domestic animals are more likely to slaughter and consume them instead of bushmeat.	[21, 47]
Crop consumption^‡^^§^	Amount of crops harvested and consumed by the household during 12 months prior to the interview (kg)	Continuous	Follow-up interview survey (2015)	Number of meals per day	Households that produce large amounts of crops are more likely to consume their harvests and avoid starvation during the crisis.	[48]
Household size^‡^^|^	Number of people living in the household	Continuous	Follow-up interview survey (2015)	Number of meals per day, bushmeat consumption, food diversity	Larger households will consume less food per capita.	[49]
Number of inhabitants^‡^^§^	Number of inhabitants at interview location	Continuous	Follow-up interview survey (2015)	Number of meals per day, bushmeat consumption, food diversity	Households in small villages that are frequently located in rural areas depend on bushmeat as a protein source, whereas households in large urban areas often consider bushmeat a delicacy.	[16, 50]
Occupation^‡^	Type of occupation: (1) agriculture and hunting, (2) industry and skilled labor, (3) services provided, and (4) unemployed and mixed categories	Categorical	Follow-up interview survey (2015)	Number of meals per day, bushmeat consumption, food diversity	Hunters and forest dwellers are more likely to continue hunting and/or consuming bushmeat during the crisis because of their proximity to wildlife and because bushmeat provides a safety net for cash income. Households with unemployed household heads are more likely to endure a reduction in their number of meals per day and food diversity.	[20, 22, 51]
Sex^‡^	Sex of respondent	Categorical	Follow-up interview survey (2015)	Number of meals per day, bushmeat consumption, food diversity	The share of meat and fish given to women may be smaller relative to adult men, whereas their share of roots and tubers is relatively large.	[48]
Age^‡^	Age of respondent	Continuous	Follow-up interview survey (2015)	Number of meals per day, bushmeat consumption, food diversity	The share of meat and fish given to children may be smaller relative to adult men, whereas their share of roots and tubers is relatively large.	[48]
Bushmeat prices^‡^^§^	Bushmeat prices (LRD)	Continuous	Follow-up interview survey (2015)	Bushmeat consumption, food diversity	Bushmeat consumption is likely to decrease if prices are relatively high.	[16, 37]
Domestic meat prices^‡^^§^	Domestic meat prices (LRD)	Continuous	Follow-up interview survey (2015)	Food diversity	Households are more likely to replace bushmeat with domestic animal meat if prices for the latter are relatively low.	[52]
Domestic meat prices*income^‡^	Interaction of domestic meat prices (LRD) with monthly income (LRD)	Continuous	Follow-up interview survey (2015)	Food diversity	The effect of income is weak where domestic meat prices are relatively low. The effect will be more pronounced where bushmeat prices are high.	[16]
Bushmeat prices*income^‡^	Interaction of bushmeat prices (LRD) with monthly income (LRD)	Continuous	Follow-up interview survey (2015)	Bushmeat consumption, food diversity	The effect of income is weak where bushmeat prices are relatively low. The effect will be more pronounced where bushmeat prices are high.	[16]

^†^Included as a test predictor

^‡^Included as a fixed-effect control predictor

^§^Variable was log-transformed to normalize skewed distribution

^|^Variable was square-root-transformed to normalize skewed distribution

To control for other potential effects that may influence dietary changes, fixed-effects control predictors were included (Table 2). Household ID, location ID, interviewer ID and interview date were included as random-effects control predictors to account for variance clustered in these groups [35].

Literacy and level of education were included as test predictors in our GLMMs because it has been shown that education may have an effect on people’s dietary choices [19]. According to the Human Development Index Report, the expected number of years of schooling for Liberians is 9.5, and adult literacy is under 43% [36]; we therefore included literacy in addition to education, in case the variability of the latter was not large enough. We expected bushmeat consumption to decrease during the Ebola crisis in households with more educated and/or literate household heads, because they may be more knowledgeable about the risks of consuming bushmeat.

Assuming that bushmeat represented the main source of animal protein in rural households before the Ebola outbreak due to its relatively low cost [19], we predicted that wealthier households would more likely decrease their consumption of bushmeat during the crisis compared to poorer households, because they could afford alternative sources of animal protein. Furthermore, if high-income households were able to replace bushmeat with alternative meat sources, then they would be less likely to experience a reduction in their number of meals per day or in the diversity of food items they consumed.

To test whether the Ebola crisis had an influence on meat prices, time period was included as a test predictor in our models. Household ID, location ID and type of meat (i.e. animal species) were included as random-effects control predictors in our models for changes in local bushmeat and domestic meat prices. Prices were collected for five different types of domestic meat, and ten different types of bushmeat (S1 Appendix). For our model assessing the change in local fish prices, location ID was included as a random-effect control predictor (type of meat and household ID were not included in the model because only prices for one type of meat were available, i.e., fish).

#### Data analysis

We analyzed the data using GLMMs and LMMs. All models were fitted in R (version 3.2.2, [53]) using the following functions of the lme4 package [54]: ‘glmer’ for GLMMs, ‘lmer’ for LMMs, and ‘glmer.nb’ for a GLMM with a negative binomial error structure. All possible random slopes components were included in the models to keep type I error rate at the nominal level of 5% [35, 55]. Numeric predictor variables were z-transformed before running the models. Collinearity among the predictor variables was assessed by computing Variance Inflation Factors (VIFs; [56]) using the function ‘vif’ of the package ‘car’ [57].

To test the overall effect of our test predictors, we compared full models to null models using likelihood ratio tests [58] in which the test predictors were omitted from the null models, and all other control fixed effects, random effects and random slopes were the same as in the full models [59]. Non-significant interactions (including the test predictors that were included as interactions) were dropped from the full model before running the final model. The significance of each test predictor was assessed by using a likelihood ratio test [35] that compared the final model to a reduced model by dropping one test predictor at a time using the R function ‘drop1’. For details on model stability, see S1 Appendix.

#### Number of meals per day

We fitted a GLMM with a Poisson error structure and log link function to investigate the change in number of meals per day. The sample size for this model was 399 households from 80 locations. Collinearity was not an issue (maximum VIF: 1.966).

#### Bushmeat consumption

We fitted two GLMMs with Poisson error structure and log link function to analyze the changes in (1) frequency of bushmeat consumption, and (2) the proportion of people in the community who preferred to eat bushmeat. The sample size for the bushmeat consumption frequency model was 277 households from 75 locations, and the sample size for the model analyzing the proportion of the community that preferred bushmeat was 267 households from 75 locations. Collinearity was not an issue (maximum VIF for bushmeat consumption frequency model: 1.929; maximum VIF for proportion of the community that preferred bushmeat model: 1.872).

#### Food diversity

We fitted two GLMMs with Poisson error structure and log link function to analyze the changes in (1) the number of different food items consumed, and (2) the number of different food groups consumed. The sample size for both models was 267 households from 73 locations. Collinearity was not an issue (maximum VIF: 1.808 for both models). With these models we analyzed one aspect of food diversity, which was the number of food items and food groups consumed, but not the change in specific foods; hence, to further investigate changes in food diversity, we also used descriptive statistics to compare the proportions of respondents that consumed each food item before and during the crisis, as well as the food groups to which these items belonged.

#### Bushmeat preference

We fitted three GLMMs with binomial error structure and logit link function to analyze the changes in the likelihood of preferring to eat three of the most frequently mentioned bushmeat species: (1) duiker, (2) monkey, and (3) pangolin. Time period was included as a test predictor and location ID as a random-effect control predictor in the models (Table 2). Monthly income, level of education, and literacy were not included as test predictors in these models because the 2010–2012 survey did not gather data on these variables. The sample size for the models was 271 households from 58 locations.

#### Meat prices

We ran two LMMs to assess whether local bushmeat and fish prices changed during the crisis. The sample size for the bushmeat prices model was 146 households from 18 locations, and included the bushmeat prices for 10 species (see S1 Appendix). The sample size for the local fish prices model was 591 households from 55 locations. The assumptions of normally distributed and homogeneous residuals were checked by visually examining a QQ-plot and the residuals plotted against the fitted values. To allow for a likelihood ratio test, the models were fitted using Maximum Likelihood (ML) rather than Restricted Maximum Likelihood (REML, [60]).

To test whether domestic meat prices changed, we ran a GLMM with a negative binomial error structure and log link function. The sample size for the model was 650 households from 58 locations, and prices were specified for 5 types of meat (see S1 Appendix). A GLMM with a negative binomial distribution was chosen based on its ability to deal with overdispersion [61]. To fit this model, prices were converted into count data by rounding them to the nearest integer.

## Results

### Number of meals per day

**Fig 2:**
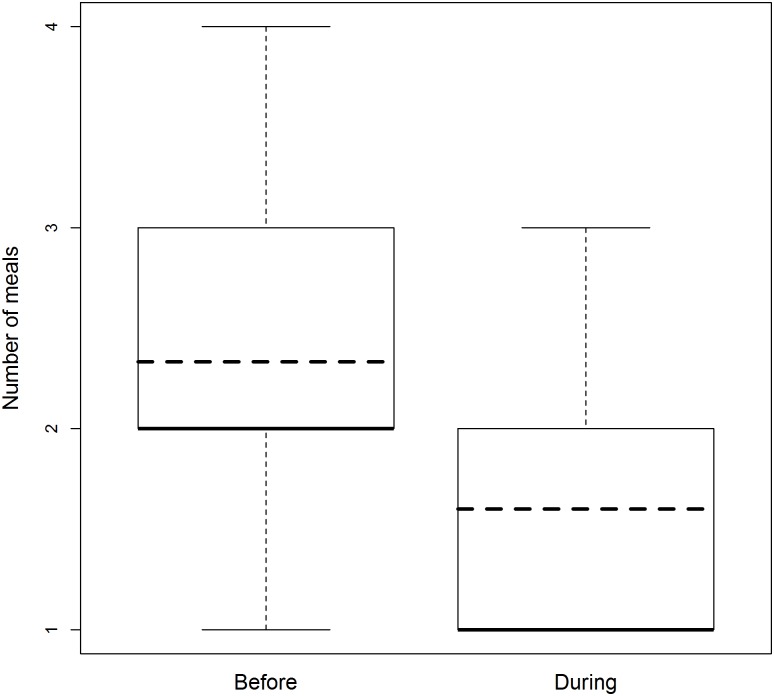
The decrease in the number of meals per day during vs. before the Ebola crisis. The bold lines show the medians, which are equal to the first quartiles for before and during the Ebola crisis, and also equal to the minimum value during the crisis. The dashed lines depict the expected values based on the model.

The full-null model comparison was significant (likelihood ratio test [LRT]: *χ*^2^ = 11.138, df = 4, p = 0.025). The number of meals that respondents consumed per day decreased significantly during the Ebola (LRT: *χ*^2^ = 10.369, df = 1, p = 0.001). Income, literacy, and the respondent’s level of education did not influence the response (Table 3, Fig 2).

**Table 3:** Estimated model coefficients and standard errors, as well as lower and upper confidence limits, degrees of freedom, p-values, and x^2^ values derived from the likelihood ratio test for the final model analyzing the change in number of meals per day.

Term	Estimate	SE	Lower CL	Upper CL	df	x^2^	p
Intercept	0.965	0.121	0.697	1.174	NA	NA	NA
Time period^‡^	-0.377	0.065	-0.486	-0.280	1	10.369	0.001
Income^§^	0.022	0.029	-0.034	0.084	1	0.549	0.459
Literacy^§^	-0.034	0.111	-0.261	0.195	1	0.094	0.760
Education^§^	0.028	0.051	-0.070	0.129	1	0.306	0.580
Perceived law enforcement^§^	0.007	0.065	-0.115	0.137	1	0.012	0.914
Ebola infections^§^	-0.051	0.063	-0.182	0.069	1	0.658	0.417
Age^§^	0.013	0.027	-0.039	0.065	1	0.228	0.633
Sex^§^	-0.110	0.072	-0.253	0.045	1	2.193	0.139
Number of inhabitants^§^	0.040	0.031	-0.021	0.104	1	1.559	0.212
Occupation, second^§^	0.108	0.097	-0.079	0.298	3	2.381	0.497
Occupation, third^§^	0.083	0.075	-0.075	0.238			
Occupation, other^§^	-0.036	0.111	-0.286	0.157			
Household size^§^	-0.026	0.027	-0.085	0.029	1	0.881	0.348
Crop consumption^§^	0.059	0.033	-0.008	0.124	1	3.286	0.070
Distance to roads^§^	<0.001	0.029	-0.063	0.052	1	<0.001	0.999
Distance to settlements^§^	-0.009	0.028	-0.063	0.041	1	0.111	0.739
Perceived risk of bushmeat consumption^§^	-0.049	0.061	-0.163	0.084	1	0.640	0.424

^‡^Included as a test predictor

^§^Included as a fixed-effects control predictor

### Bushmeat consumption

**Fig 3:**
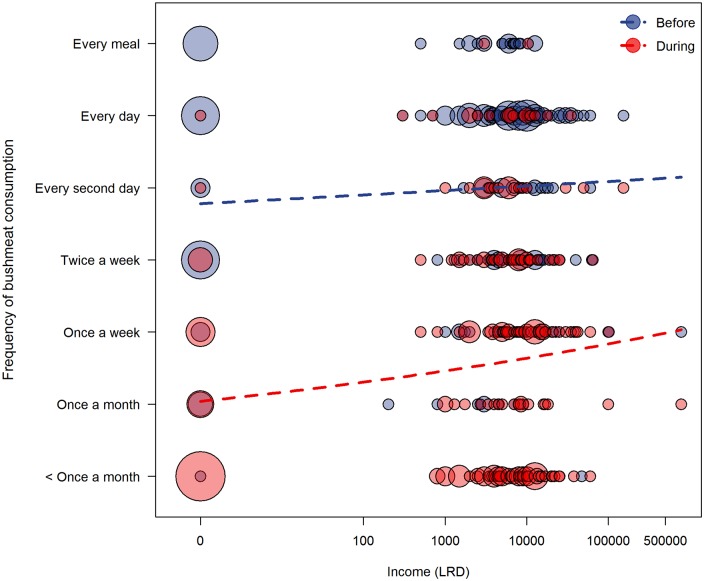
The effect of household income on bushmeat consumption frequency before vs. during the crisis. The size of each circle corresponds to the proportion of households and the dashed lines depict the fitted regressions for each time period.

The full model testing for changes in frequency of bushmeat consumption fitted the data significantly better than its corresponding null model (LRT: *χ*^2^ = 21.029, df = 4, p <0.001). The interaction of time period with income was a trend (LRT: *χ*^2^ = 3.119, df = 1, p = 0.077; Table 4, Fig 3). In addition, the control predictor for local bushmeat prices was a trend (LRT: *χ*^2^ = 3.813, df = 1, p = 0.051; Table 4), indicating a decrease in bushmeat consumption frequency where local bushmeat prices were high. Only the control predictor for perceived risk of bushmeat consumption had a significant influence on the frequency of bushmeat consumption; households were more likely to decrease their consumption frequency if the household head believed that Ebola could be contracted from bushmeat (LRT: *χ*^2^ = 8.731, df = 1, p = 0.003; Table 4).

**Table 4:** Estimated model coefficients and standard errors, as well as lower and upper confidence limits, degrees of freedom, p-values, and x^2^ values derived from the likelihood ratio test for the final model analyzing the change in the frequency of bushmeat consumption.

Term	Estimate	SE	Lower CL	Upper CL	df	x^2^	p
Intercept	1.521	0.120	1.166	1.826	NA	NA	NA
Time period*income^‡^	0.147	0.083	-0.076	0.392	1	3.119	0.077
Literacy^§^	0.052	0.112	-0.258	0.357	1	0.211	0.646
Education^§^	-0.049	0.053	-0.208	0.089	1	0.814	0.367
Perceived law enforcement^§^	-0.089	0.065	-0.285	0.102	1	1.798	0.180
Ebola infections^§^	0.035	0.070	-0.164	0.230	1	0.246	0.620
Age^§^	-0.031	0.029	-0.109	0.047	1	1.069	0.301
Sex^§^	0.041	0.086	-0.189	0.288	1	0.216	0.642
Number of inhabitants^§^	-0.087	0.053	-0.233	0.061	1	2.238	0.135
Occupation, second^§^	-0.034	0.107	-0.364	0.260	3	1.746	0.627
Occupation, third^§^	0.007	0.069	-0.196	0.184			
Occupation, other^§^	-0.152	0.119	-0.513	0.178			
Household size^§^	0.006	0.033	-0.091	0.093	1	0.031	0.861
Livestock consumption^§^	0.051	0.037	-0.049	0.150	1	1.605	0.205
Distance to roads^§^	0.012	0.031	-0.084	0.103	1	0.123	0.726
Distance to settlements^§^	0.008	0.029	-0.073	0.083	1	0.076	0.783
Perceived risk of bushmeat consumption^§^	-0.197	0.064	-0.368	-0.025	1	8.731	0.003
Bushmeat prices^§^	-0.067	0.033	-0.152	0.027	1	3.813	0.051

^‡^Included as a test predictor

^§^Included as a fixed-effects control predictor

**Fig 4:**
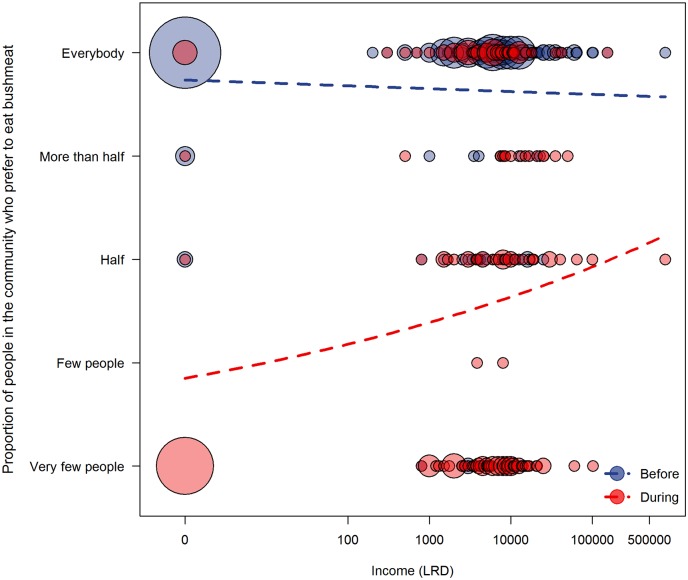
The effect of household income on the proportion of people in the community that consumed bushmeat before vs. during the crisis. The size of each circle corresponds to the proportion of households and the dashed lines depict the fitted regressions for each time period.

We found a similar pattern regarding changes in the proportion of the community that preferred to eat bushmeat. There was a significant difference between the full model and its respective null model (LRT: *χ*^2^ = 12.964, df = 4, p = 0.012). The interaction of time period with income had a significant effect (LRT: *χ*^2^ = 4.073, df = 1, p = 0.044; Table 5, Fig 4). Heads of low-income households thought that a smaller proportion of their community continued to prefer eating bushmeat during the crisis, whereas heads of high-income households perceived a smaller decrease in bushmeat consumption in their community.

**Table 5:** Estimated model coefficients and standard errors, as well as lower and upper confidence limits, degrees of freedom, p-values, and x^2^ values derived from the likelihood ratio test for the final model analyzing the change in the proportion of the community that preferred to eat bushmeat.

Term	Estimate	SE	Lower CL	Upper CL	df	x^2^	p
Intercept	1.161	0.134	0.784	1.505	NA	NA	NA
Time period*income^‡^	0.256	0.117	-0.110	0.585	1	4.073	0.044
Literacy^§^	0.045	0.116	-0.271	0.371	1	0.145	0.703
Education^§^	-0.038	0.054	-0.179	0.105	1	0.493	0.483
Perceived law enforcement^§^	-0.024	0.071	-0.214	0.195	1	0.113	0.737
Ebola infections^§^	0.062	0.074	-0.154	0.266	1	0.690	0.406
Age^§^	-0.004	0.031	-0.087	0.075	1	0.018	0.894
Sex^§^	0.144	0.097	-0.122	0.418	1	1.833	0.176
Number of inhabitants^§^	-0.049	0.035	-0.149	0.044	1	2.012	0.156
Occupation, second^§^	0.004	0.108	-0.334	0.287	3	0.292	0.962
Occupation, third^§^	-0.006	0.075	-0.203	0.203			
Occupation, other^§^	-0.084	0.157	-0.520	0.353			
Household size^§^	0.021	0.031	-0.060	0.100	1	0.374	0.541
Livestock consumption^§^	0.006	0.030	-0.088	0.084	1	0.034	0.853
Distance to roads^§^	<0.001	0.035	-0.093	0.088	1	<0.001	0.981
Distance to settlements^§^	0.003	0.032	-0.084	0.089	1	0.009	0.926
Perceived risk of bushmeat consumption^§^	-0.009	0.070	-0.200	0.176	1	0.195	0.659
Bushmeat prices^§^	-0.008	0.035	-0.102	0.091	1	0.155	0.694

^‡^Included as a test predictor

^§^Included as a fixed-effects control predictor

### Food diversity

**Fig 5:**
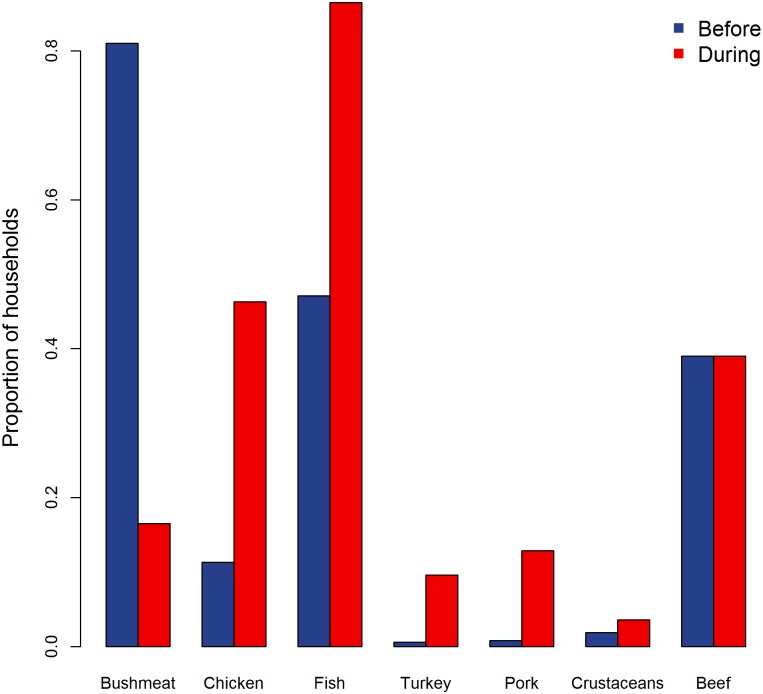
The change in the proportion of households consuming different types of meat in a typical meal before and during the Ebola crisis.

Both full models testing for changes in food diversity were not different from their respective null models (change in number of food items consumed, LRT: *χ*^2^ = 3.841, df = 4, p = 0.428; change in number of food groups consumed, LRT: *χ*^2^ = 2.411, df = 4, p = 0.661). As a complement to our models, we used descriptive statistics to further investigate changes in food diversity, and we found differences in the proportions of individual food items and food groups that were typically consumed in a meal before and during the crisis. Notably, the consumption of bushmeat dropped from 81% to 16.5%, while chicken and fish consumption increased from 11.3% to 46.3% and from 47.1% to 86.5% respectively (Table 6, Fig 5).

**Table 6:** The proportions of households that consumed each of the most commonly mentioned food items and food groups during a typical meal on a typical day before and during the Ebola crisis.

**Food items in a typical meal before the Ebola crisis**											
Staples	0.906	Vegetables	0.265	Fruits	0.185	Meat	0.950	Fish and seafood	0.471	Oils	0.377
Rice	0.893	Cassava leaves	0.033	Banana	0.168	Bushmeat	0.810	Fish	0.471		
Cassava	0.377	Eddoes	0.110	Breadfruit	0.030	Chicken	0.113	Crustaceans	0.019		
Plantain	0.223	Potato greens	0.036	Coconut	0.028	Beef	0.039				
Potato	0.052	Bitter root	0.025			Pork	0.008				
		Bitter balls	0.025			Turkey	0.006				
**Food items in a typical meal during the Ebola crisis**											
Staples	0.843	Vegetables	0.220	Fruits	0.121	Meat	0.620	Fish and seafood	0.865	Oils	0.237
Rice	0.810	Cassava leaves	0.017	Banana	0.058	Bushmeat	0.165	Fish	0.865		
Cassava	0.251	Eddoes	0.041	Breadfruit	0.050	Chicken	0.463	Crustaceans	0.036		
Plantain	0.135	Potato greens	0.006	Coconut	0.039	Beef	0.039				
Potato	0.006	Bitter root	0.129			Pork	0.129				
		Bitter balls	0.014			Turkey	0.096				

### Bushmeat preference

None of the three full models were different from their respective null models (preference for monkey meat, LRT: *χ*^2^ = 2.317, df = 1, p = 0.128; duiker meat, LRT: *χ*^2^ = 0.525, df = 1, p = 0.469; and pangolin meat, LRT: *χ*^2^ = 0.106, df = 1, p = 0.74). Thus, the likelihood of respondents preferring to eat these three species during the Ebola crisis did not change.

### Meat prices

The full model for changes in local bushmeat prices was not significantly different from the null model (LRT: *χ*^2^ = 2.260, df = 1, p = 0.133); hence, prices for bushmeat did not significantly change during the Ebola crisis. Fish prices significantly increased during the crisis (Table 7) by 19.5%, from an estimated 71.9 to 85.9 Liberian dollars per piece (LRT: *χ*^2^ = 4.804, df = 1, p = 0.028).

**Table 7:** Estimated model coefficients and standard errors, lower and upper confidence limits, degrees of freedom, p-values, and x^2^ values derived from the likelihood ratio tests for the final models analyzing the change in fish and domestic meat prices.

Model/response	Term	Estimate	SE	Lower CL	Upper CL	df	x^2^	p
Fish prices	Intercept	4.275	0.073	4.075	4.470	NA	NA	NA
	Time period	0.178	0.080	-0.041	0.399	1	4.804	0.028
Domestic meat prices	Intercept	8.232	0.732	6.226	10.199	NA	NA	NA
	Time period	0.296	0.062	0.121	0.482	1	8.864	0.003

Likewise, domestic meat prices increased during the crisis (Table 7) by 34%, from 3757.0 to 5050.0 Liberian dollars per body (LRT: *χ*^2^ = 8.864, df = 1, p = 0.003).

## Discussion

People in Liberia consumed less bushmeat during the EVD crisis than before. However, our study suggests that wealthier people reduced their consumption of bushmeat less than those with a lower income. People also suffered from food shortages as they ate fewer meals per day during, as compared to before the crisis. Although the diversity of food items that made up people’s meals did not change, it seems likely that the items themselves changed and bushmeat was mainly replaced by chicken and fish. However, people’s preference for specific bushmeat species remained the same.

There was a clear effect of the Ebola crisis on daily meal frequency, which decreased across all levels of income and education. Several factors could have contributed to this food shortage; quarantine measures and border closures greatly restricted the movement of people and goods, disrupting agricultural activities and businesses, leading to aggravated food insecurity [47, 62]. Furthermore, the areas with a high incidence of EVD infections were also the most productive regions in the country, where a shortage of labor during the crisis caused a drop in both food and cash-crop production [63]. Consequently, household incomes were negatively impacted and food accessibility was further inhibited [64]. In addition, the limited distribution of imported foods from Monrovia’s sea-port to rural markets during the crisis resulted in price increases of some food items [63].

Our study shows that the frequency of bushmeat consumption during the Ebola crisis was influenced by household income. In addition, we found in our control predictors an influence of (1) bushmeat prices, and (2) the perceived risk of bushmeat consumption. We interpreted these as two components of the costs associated with bushmeat consumption: (1) the monetary costs and (2) the health risk costs. Bushmeat price is therefore not only monetary, but also contains a health component. Both poor and rich households were subject to health risk costs, and people were therefore more likely to consume bushmeat less frequently if they thought that EVD could be contracted from bushmeat. This may partly explain the overall decrease in bushmeat consumption during the crisis in Liberia across all levels of household income. However, the monetary costs had a greater influence on the consumption habits of poor households, which were more affected by the prices of bushmeat compared to wealthy households, as the latter could compensate higher prices with higher incomes. This coincides with the economic law of demand, which predicts that the demand for a good decreases if the price for it increases [65].

The demand for bushmeat is partially driven by bushmeat prices and the prices of similar substitutes [52]. This dynamic is also supported by our model results on individually reported bushmeat consumption frequency: bushmeat consumption decreased with increasing prices. To further investigate the changes in bushmeat consumption, we therefore tested whether prices for domestic meats and bushmeat changed between the two surveys. We found that fish and domestic meats became more expensive during the crisis, which can be explained by an increase in their demand (reflected in our food diversity analysis) but a decrease or stability in their production [66], and possibly also a limited accessibility to them due to the travel restrictions during the crisis [62] and the high costs of transportation to some parts of the country [19]. In contrast, our results indicated that prices for bushmeat remained stable during the crisis. This finding is puzzling and does not match the model results or theoretical predictions. However, a likely explanation is that this price stability was caused by opposing trends in bushmeat demand and bushmeat hunting that may have occurred as a result of the EVD crisis. A very likely decrease in hunting rates during the crisis would result in a reduced supply, which would presumably increase bushmeat prices; however, the much lower demand for bushmeat during the crisis (demonstrated by the decrease in bushmeat consumption) may have counteracted this effect, resulting in price stability. An additional explanation may be that our sample size on bushmeat prices was small. This may have complicated the detection of a change in bushmeat prices.

Our analysis for the change in the proportion of people in the community that preferred to eat bushmeat showed a similar effect of income on bushmeat consumption. However, this model was problematic due to the difference in scale of the response and the predictors; i.e., the response was at the community level, but the predictors were at the household level. Hence, our interpretation of this model is that the perception of respondents about the consumption of bushmeat in the community was influenced by their income level. We do not intend to interpret the other predictors in the model due to the described problem of scale in the model.

The number of food groups and food items consumed remained constant during the crisis; nevertheless, there were differences in diet composition, reflected by the proportions of different food items and food groups consumed. Our results suggest that chicken and fish were important substitutes for bushmeat for Liberians during the Ebola crisis.

The drop in bushmeat consumption during the crisis does not imply that people did not like bushmeat anymore; indeed, the likelihood of respondents preferring to eat duiker, pangolin or monkey meat did not change. This finding indicates that factors such as taste preference or tradition may play an influential role in human dietary choices. This may have important implications for future conservation management. For example, if taste preference is a major driver of bushmeat consumption, the demand for bushmeat may be less responsive to price changes, and finding an acceptable substitute may be difficult [67]. Furthermore, due to the consistent preference for bushmeat during the crisis, people may continue to consume it as soon as the memories of the impacts of the crisis have faded sufficiently.

### Implications for conservation

The overall decrease in bushmeat consumption associated with the Ebola crisis may have had a short-term positive effect on vulnerable wildlife populations. Needless to say, however, that this should not advocate the use of fear for the disease as a medium for accomplishing conservation goals [68]. It is problematic to suggest that the epidemic presented a “silver lining” for conservation [69] because of the catastrophic impacts on human livelihoods and food security. Furthermore, spreading fear about the disease could backfire in that this may lead to attempts to eradicate the vectors of the deadly disease [68].

Conservation efforts should instead focus on developing strategies that are compatible with human livelihoods and food security [18]. Due to the complexity and variability of bushmeat consumption drivers that need to be addressed, multiple interventions may be required [51, 70]. Based on our results, conservation strategies that aim to reduce bushmeat consumption in Liberia may be more effective by making a distinction between the consumption patterns of high-income households to those of low-income households. In addition, although we did not find an effect of literacy or education on bushmeat consumption during the Ebola crisis, environmental education should not be disregarded as an important component in conservation strategies, as it has been shown to correlate with environmental health [19, 71]. Indeed, household heads were more likely to consume bushmeat less frequently if they thought that Ebola could be contracted from bushmeat, suggesting that knowledge about the disease had an impact on bushmeat consumption patterns. This also means that there is a difference in the effect of general education and specific knowledge on human behavioural patterns.

Law enforcement is often called for as an indispensable means of mitigating the illegal bushmeat trade (e.g., [70, 72, 73]). The enforcement of laws that prohibit the sale and consumption of protected and endangered species in urban markets is crucial for reducing the demand for bushmeat of high-income, urban households [18]. However, a complete ban on bushmeat is unrealistic, given that poor households that rely heavily on bushmeat as a source of nutrition will be negatively impacted; especially if alternative sources of protein are not provided [49, 70, 74]. It is therefore important to distinguish between resilient species that may still be hunted sustainably from those that are too vulnerable to be harvested [20].

Providing alternative income and protein sources could reduce the reliance on bushmeat of low-income households [19, 70, 75]. Similar to other studies [17, 19, 76] our results indicate that fish is an important alternative protein source to bushmeat. However, if fish represents a direct replacement for bushmeat, it is necessary to improve the management of domestic fisheries to help increase the sustainability of fish stocks [76,77]. Similarly, meat from domestic animals may be an acceptable replacement for bushmeat; however, the negative environmental impacts associated with increased livestock production must be reduced through proper management [70, 77]. Furthermore, it is also important to secure the availability of staple foods such as grains, roots and tubers throughout the year [70] and expand the use of plant proteins, such as dried beans and other pulses, which have a long shelf life when stored properly and could provide a readily-available source of protein in times of crisis [78].

## Supporting information

S1 Appendix Details on data preparation for response and predictor variables, and model stability. (PDF)

S2 Appendix Complete questionnaires used during the 2010–212 and 2015 Liberia nationwide surveys. (PDF)

S1 Data The dataset contains all interview data used in our analysis that were collected from 623 Liberian households during the 2015 survey. (XLSX)

S2 Data The dataset contains all interview data used in our analysis that were collected from 275 Liberian households during the 2010–2012 survey. (XLSX)
