# Supplementary material for: The socio-economic drivers of bushmeat consumption during the West African Ebola crisis
Source: PLoS Negl Trop Dis. 2017 Mar 10;11(3):e0005450. doi: 10.1371/journal.pntd.0005450 (PMC5362244; doi:10.1371/journal.pntd.0005450)
Supplement: S1 Appendix — (PDF) [file pntd.0005450.s001.pdf]

S1.1 Data preparation for response variables

Frequency of bushmeat consumption

Respondents could choose among eight categories to specify how frequently they consumed bushmeat: 'with every meal', 'every day', 'every second day', 'twice a week', 'once a week', 'once a month', '< once a month', 'never'. We reduced the number of levels by moving the number of respondents who said 'never' (n = 210) to '< once a month' because the level 'never' was absent as an optional response to the retrospective question in the questionnaire; hence, we needed to match the response levels between the two time periods. The categories were first transformed into ordered factors, from the lowest to the highest frequency of consumption, and then into ranks.

Proportion of people in the community who preferred to eat bushmeat

Respondents could choose among five options to specify what proportion of people in their community or nearby communities they thought preferred to eat bushmeat before and during the Ebola crisis: 'very few people', 'half', 'more than half', 'everybody', and 'other'. We moved the number of respondents who selected the option 'other' and then specified 'nobody', to the level called 'very few people', as it was the most proximate level. We created an additional level for respondents who selected the option 'other' and then specified 'few people' because a level between 'very few people' and 'half' was absent. The categories were first transformed into ordered factors, from the lowest to the highest proportion of the community who preferred to eat bushmeat, and then into ranks.

Number of food items consumed

Respondents provided lists of food items that composed a typical meal on a typical day before and during the Ebola outbreak, which were transformed into count data to investigate changes in food diversity.

Number of food groups consumed

We used the lists of food items that described a typical meal on a typical day before and during the crisis to create six food groups: staples, vegetables, fruits, meat, fish and seafood, and oil. To investigate changes in food diversity based on the number of food groups consumed, we converted these into count data.

Bushmeat preference

Respondents were asked 'if there was a festivity to which you were invited, which type of meat would you prefer to eat?' if the answer was 'bushmeat', they were asked to specify the species in the consecutive question. We selected three of the most commonly preferred species (duikers, pangolins and monkeys), and ran separate models for each one. For each model we converted the ordinal data into binary data (e.g. pangolin was given the value of one, and all other bushmeat species the value of zero) to compare the likelihood of preference for each species before and during the crisis.

Meat prices

Fish, bushmeat and domestic meat prices that were specified in US dollars were converted into Liberian dollars (LRD). The exchange rate at the time of the 2015 follow-up survey was 84 LRD for 1 USD [1].

S1.2 Data preparation for predictor variables

Distance to roads and distance to settlements

To control for an effect of the remoteness of interview locations we created two raster layers in ArcGIS [2], one for settlements and one for roads. Within each layer we calculated the Euclidean distance from each pixel to the closest road or settlement. The layers were converted into ASCI files, which contained the distance values for each pixel and were used for the statistical analysis in R [3]. To assess whether the two predictor variables were correlated we calculated the Spearman's rank correlation coefficient that showed only a weak correlation between the two variables (Spearman's rho= 0.247).

Occupation

We divided the types of occupation into four categories: 'first' for agriculture and hunting, 'second' for industry and skilled labor, 'third' for services provided, and 'other' for unemployed or mixed categories.

Livestock consumption

To obtain the amount of livestock and domestic animals (in kg) that were owned and consumed by the household, we multiplied the number of each type of animal slaughtered during the previous year with the average weight of the animal.

Crop consumption

To estimate the amount of crops consumed by the household (in kg), we took the total production from the previous year and subtracted the amount reported for the total production that was sold. We assumed that this amount was consumed by the household.

Household size

To control for household size, we summed the number of male and female members of the household from all age groups.

Bushmeat and domestic meat prices

Meat prices that were specified in US dollars were converted into Liberian dollars (LRD). The exchange rate at the time of the 2015 follow-up survey was 84 LRD for 1 USD [1].

Meat types

We collected prices for five types of domestic meat: chicken, beef, pork, sheep, and goat. For bushmeat, prices were given for 10 types of meat: monkey, porcupine, cane rat, water chevrotain, crocodile, antelope, red river hog, bay duiker, black duiker, and Maxwell's duiker.

S1.3 Model stability

We assessed model stability by re-running the full models that excluded levels of the random effects one at a time, and comparing the estimated coefficients from the reduced models to the ones obtained from a model based on the full data set.

Table S1.1 Original estimates, lower- and upper bounds of coefficients for each test predictor for models based on the full data set.

Model/response	Predictor	Original estimate	Lower bound	Upper bound
Number of meals per day	Intercept	0.964	0.949	-0.991
	Time period†	-0.373	-0.466	-0.218
	Time period*income†	0.045	0.029	0.08
	Time period*literacy†	-0.008	-0.149	0.103
	Time period*education†	0.011	-0.044	0.073
	Income‡	0.004	-0.009	0.019
	Literacy‡	-0.031	-0.055	0.001
	Education‡	0.024	-0.001	0.037
	Perceived law enforcement‡	0.006	-0.016	0.017
	Ebola infections‡	-0.052	-0.094	-0.015
	Age‡	0.013	-0.004	0.025
	Sex‡	-0.109	-0.137	-0.083
	No. of inhabitants‡	0.04	0.024	0.052
	Occupation, other‡	-0.033	-0.063	-0.014
	Occupation, second‡	0.109	0.059	0.14
	Occupation, third‡	0.084	0.051	0.127
	Household size‡	-0.026	-0.036	-0.016
	Crop consumption‡	0.059	0.04	0.071
	Distance from settlements‡	-0.01	-0.022	0.001
	Distance from roads‡	0.000	-0.016	0.019
	Bushmeat perception‡	-0.049	-0.069	-0.035

Frequency of bushmeat consumption	Intercept	1.515	1.432	1.575
	Time period[†]	-0.919	-1.070	-0.851
	Time period*income[†]	0.166	0.093	0.190
	Time period*literacy[†]	-0.080	-0.205	0.134
	Time period*education[†]	-0.050	-0.111	0.001
	Income[‡]	0.024	-0.009	0.055
	Literacy[‡]	0.075	0.017	0.162
	Education[‡]	-0.035	-0.064	-0.019
	Perceived law enforcement[‡]	-0.093	-0.119	-0.068
	Ebola infections[‡]	0.032	-0.006	0.086
	Age[‡]	-0.029	-0.053	-0.015
	Sex[‡]	0.029	-0.012	0.099
	No. of inhabitants[‡]	-0.091	-0.112	-0.057
	Occupation, other[‡]	-0.143	-0.299	-0.082
	Occupation, second[‡]	-0.036	-0.105	0.017
	Occupation, third[‡]	0.005	-0.016	0.030
	Household size[‡]	0.003	-0.034	0.046
	Livestock consumption[‡]	0.051	0.017	0.072
	Distance from settlements[‡]	0.006	-0.006	0.037
	Distance from roads[‡]	0.011	-0.001	0.033
	Bushmeat perception[‡]	-0.194	-0.263	-0.148
	Bushmeat prices[‡]	-0.194	-0.082	-0.052
	Income*bushmeat prices[‡]	0.017	0.000	0.033
Proportion of people in the community who preferred to eat bushmeat	Intercept	1.182	1.105	1.359
	Time period[†]	-1.119	-1.553	-0.884
	Time period*income[†]	0.293	0.240	0.581
	Time period*literacy[†]	0.249	0.140	0.355
	Time period*education[†]	-0.206	-0.268	-0.150
	Income[‡]	-0.023	-0.036	-0.004
	Literacy[‡]	-0.018	-0.047	0.019
	Education[‡]	0.002	-0.015	0.013
	Bushmeat prices[‡]	-0.009	-0.026	0.023
	Perceived law enforcement[‡]	-0.015	-0.057	0.026
	Ebola infections[‡]	0.066	0.036	0.088
	Age[‡]	-0.006	-0.020	0.002
	Sex[‡]	0.160	0.079	0.246
	No. of inhabitants[‡]	-0.048	-0.061	-0.026
	Occupation, other[‡]	-0.099	-0.176	0.031
	Occupation, second[‡]	0.010	-0.024	0.060
	Occupation, third[‡]	-0.004	-0.041	0.015
	Household size[‡]	0.024	0.006	0.036
	Livestock consumption[‡]	0.003	-0.003	0.022
	Distance from settlements[‡]	0.004	-0.028	0.024
	Distance from roads[‡]	-0.001	-0.009	0.021
	Bushmeat perception[‡]	-0.006	-0.046	0.035
	Income*bushmeat prices[‡]	-0.042	-0.060	-0.031

Number of food items consumed	Intercept	1.408	1.250	1.466
	Time period†	-0.156	-0.242	-0.083
	Time period*income†	0.035	0.017	0.066
	Time period*literacy†	0.033	-0.008	0.127
	Time period*education†	-0.059	-0.097	-0.041
	Income‡	0.046	-0.024	0.094
	Literacy‡	0.085	0.031	0.165
	Education‡	0.005	-0.044	0.038
	Bushmeat prices‡	0.041	0.031	0.049
	Domestic meat prices‡	-0.037	-0.063	-0.014
	Perceived law enforcement‡	0.006	-0.006	0.034
	Ebola infections‡	0.008	-0.025	0.033
	Age‡	-0.002	-0.019	0.039
	Sex‡	-0.048	-0.092	-0.020
	No. of inhabitants‡	0.000	-0.012	0.023
	Occupation, other‡	0.076	0.040	0.139
	Occupation, second‡	0.048	0.027	0.092
	Occupation, third‡	0.082	0.044	0.103
	Household size‡	0.010	-0.003	0.018
	Distance from settlements‡	0.004	-0.005	0.022
	Distance from roads‡	0.013	-0.019	0.035
	Bushmeat perception‡	-0.061	-0.079	-0.038
	Income*bushmeat prices‡	-0.018	-0.029	0.001
	Income*domestic prices‡	0.021	0.009	0.032
Number of food groups consumed	Intercept	1.191	1.106	1.273
	Time period†	-0.045	-0.109	0.012
	Time period*income†	-0.014	-0.030	0.011
	Time period*literacy†	-0.038	-0.086	0.007
	Time period*education†	-0.011	-0.034	0.007
	Income‡	0.062	0.000	0.132
	Literacy‡	0.056	0.036	0.093
	Education‡	-0.016	-0.052	-0.006
	Bushmeat prices‡	0.036	0.028	0.051
	Domestic meat prices‡	-0.031	-0.062	-0.014
	Perceived law enforcement‡	0.017	-0.035	0.085
	Ebola infections‡	-0.007	-0.063	0.019
	Age‡	0.000	-0.008	0.022
	Sex‡	-0.118	-0.173	-0.043
	No. of inhabitants‡	0.012	0.000	0.038
	Occupation, other‡	-0.020	-0.062	0.029
	Occupation, second‡	-0.024	-0.046	0.030
	Occupation, third‡	0.001	-0.022	0.019
	Household size‡	0.014	0.002	0.022
	Distance from settlements‡	-0.006	-0.019	0.007
	Distance from roads‡	0.019	0.003	0.057
	Bushmeat perception‡	-0.008	-0.029	0.015
	Income*bushmeat prices‡	-0.024	-0.040	-0.012
	Income*domestic prices‡	0.014	0.006	0.019

Preference for duiker meat	Intercept<br>Time period†	-1.454<br>0.278	-1.694<br>0.000	0.000<br>0.360
Preference for monkey meat	Intercept<br>Time period†	-2.512<br>-0.916	-2.734<br>-1.047	0.000<br>0.000
Preference for pangolin meat	Intercept<br>Time period†	-1.599<br>-0.121	-1.757<br>-0.223	-1.524<br>0.025
Domestic meat prices	Intercept<br>Time period†	4.275<br>0.179	4.252<br>0.148	4.304<br>0.214
Bushmeat prices	Intercept<br>Time period†	6.844<br>0.155	6.704<br>0.087	6.96<br>0.253
Fish prices	Intercept<br>Time period†	4.275<br>0.179	4.252<br>0.148	4.304<br>0.214

†Included as a test predictor
‡Included as a fixed-effect control predictor

References

1. Oanda Currency Converter. Available from: http://www.oanda.com/currency/converter/
2. Environmental Systems Research Institute (ESRI). ArcGIS Desktop: Release 10.1. 2011. Redlands.
3. R Core Team. R: A language and environment for statistical computing. Vienna: R Foundation for Statistical Computing; 2015. Available from: https://www.R-project.org/
