# Supplementary material for: The socio-economic drivers of bushmeat consumption during the West African Ebola crisis
Source: PLoS Negl Trop Dis. 2017 Mar 10;11(3):e0005450. doi: 10.1371/journal.pntd.0005450 (PMC5362244; doi:10.1371/journal.pntd.0005450)
Supplement: S2 Appendix — (PDF) [file pntd.0005450.s002.pdf]

1

FOLLOW-UP OF AFRICA WIDE GREAT APE POPULATION SURVEILLANCE / LIBERIA 2015

2

N°

IMPORTANT: select interviewees **RANDOMLY** whenever possible; **NEVER** interview the same person that was interviewed by us before in the 2010-2012 survey; interview **the same person** for both data sheets; interview **household heads** whenever possible, circle answer for **SINGLE** and **ANY** questions)

I. PART ONE: BASIC INFORMATION ON LOCATION & RESPONDENT

1. Location name (OPEN)____
2. Location type (SINGLE: village, logging camp, mining camp, other)

If other ,what? (OPEN)

3. Estimated number of inhabitants (NUM, inhabitants)
4. GPS coordinates (NUM, UTM coordinates, geographically bounded)

Longitude ______________________Latitude__________________________

5. Date (NUM, date)
6. Start time (NUM, time)
7. Interviewer (OPEN)____

8.a) How was the respondent selected? (SINGLE: randomly selected, you sought them out following another's suggestion, they volunteered themselves, other)

8.b) If other, how? (OPEN)____

9. Age (NUM, years)
10. Sex (SINGLE: female, male)
11. Ethnic background (OPEN)____
12. **Literacy: Can you read or write a sentence in the language/dialect of your choice? (SINGLE: yes, no)**

13.a) How many years of 1° education did you complete (NUM, years)?

13.b) How many years of 2° education did you complete (NUM, years)?

14. How many years have you lived here? (NUM, years)

15.a) Religion (SINGLE: Muslim, Christian, Hindu, Buddhist, atheist, other)

15.b) If other, what? (OPEN) ____________________________________

16.a) Does your religion forbid the killing or eating of any animal species? (SINGLE: yes, no, don't know)

16.b) If yes, killing, eating or both? (SINGLE: killing, eating, both)

16.c) If yes, which species? (OPEN)__________________________________

____

II. PART TWO: BUSHMEAT CONSUMPTION & HUNTING PRACTICE

17.a) If there was a festivity (party) to which you were invited, which type of meat would you prefer to eat? (SINGLE: chicken, pig, bush meat, horse, donkey, goat, cane rats, fish, sheep, snails, beef, guinea pig, pigeon, other)

17.b) If other, what? (OPEN)____

17.c) If bush meat, which animal? (OPEN)_________________________________

17.d) Why do you prefer this meat? (ANY: price, availability, tastes good, healthy, meat quality, tradition/culture, local beliefs (for example, "it makes me strong"), other)

17.e) If other, what? (OPEN)____

18.a) Do you know of a forest near your community? (SINGLE: yes, no)

18.b) If yes, what is the name of the forest? (OPEN)____________________________

18.c) If yes, how often do you go into the forest? (SINGLE: 5 or >5 times a week, 2-4 times a week, 1-2 times a week, 1-2 times a month, 1-2 times a year, never)

18.d) If answer ≠ never, when you go to the forest, do you spend the night there? (SINGLE: yes, no, sometimes)

18.e) If yes, or sometimes, how many nights per trip? (NUM, nights)

18.f) If answer ≠ never, when was the last time you went to the forest (SINGLE: yesterday, within the last 7 days, within the last 30 days, within the last 6 months, within the last year)

18.g) If answer ≠ never, what do you go to the forest for? (ANY: timber, non-timber forest products, honey, mining, hunting, traditional medicine, other)

18.h) If other, what? (OPEN)_______________________________________

18.i) If answer = hunt, where do you usually hunt? (NUMBER: distance from your home in km ___________; in hrs ____________)

18.j) If = hunt, which animals do you most commonly manage to hunt? (OPEN)____

____

18.k) If answer = hunt, what tools do you use to hunt? (ANY: snare, guns, dogs & gun, others)

18.l) If answer = hunt, do you usually hunt at night or during the day (SINGLE: night, day, both)

18.m) If answer = hunt, are there particular times of the year where you hunt (more)? (SINGLE: yes, no, don't know)

18.n) If yes, which months/time of the year do you hunt less? (OPEN)____

18.o) If yes, why? (OPEN)____________________________________

____

III. PART THREE: LAW ENFORCEMENT

19.a) Are any animal species protected by local taboos in your community? (SINGLE: yes, no, don't know)

19.b) If yes, which one(s)? (OPEN)___________________________

____

19.c) If yes, who taught you this? (SINGLE: village chief, elder, school teacher, family, friend, colleague, I taught myself, other)

19.d) If other, who? (OPEN)_________________________________

20.a) Have any new taboos been implemented in your community because of the Ebola crisis? (SINGLE: yes, no, don't know)

20.b) If yes, which ones? (OPEN)____________________________

____

____

20.c) If yes, why? (OPEN)__________________________________

____

21.a) Have any local taboos been cancelled/lifted because of the Ebola crisis?(SINGLE: yes, no, don't know)

21.b) If yes, which ones? (OPEN)____________________________

____

21.c) If yes, why? (OPEN)__________________________________

____

22.a) Are any animal species protected by Liberian law? (SINGLE: yes, no, don't know)

22. b) If yes, which ones (OPEN)?__________________________________

____

22.c) If yes, who taught you this? (SINGLE: FDA official, conservation organization, school teacher, development firm, other organization, village chief, elder, family, friend, colleague, I taught myself, other)

22.d) If other, what? (OPEN)_________________________________

____

22.e) If answer =conservation organization, =development firm, =other organization, what is the name of the organization/firm? (OPEN) ____

23.a) Do you think that the laws to protect animals were effectively implemented **BEFORE the Ebola crisis?** (SINGLE: yes, no, don't know)

24.a) Do you know of people that were punished by the law for killing animals? (SINGLE: yes, no, don't know)

FOLLOW-UP OF AFRICA WIDE GREAT APE POPULATION SURVEILLANCE / LIBERIA 2015

3 4

N°

24.b) If yes, how many? (NUM, no. people punished)

24.c) If yes, how were they punished? (ANY: fine, jail, officially warned, other)

24.d) If other, what? (OPEN)____

24.e) If yes, by whom? (OPEN)____

25.a) Do you think that the laws to protect animals are effectively implemented **NOW during the Ebola crisis?** (SINGLE: yes, no, don't know)

25.a) Do you know of people that were punished by the law for killing animals **NOW during the Ebola crisis?**? (SINGLE: yes, no, don't know)

25.b) If yes, how many? (NUM, no. people punished)

25.c) If yes, how were they punished? (ANY: fine, jail, officially warned, other)

25.d) If other, what? (OPEN)____

25.e) If yes, by whom? (OPEN)____

26.a) Is there a special ban against hunting bushmeat due to the Ebola crisis? (SINGLE: yes, no, don't know)

26.b) If yes, against which species? (OPEN)________________________________

____

26.c) If yes, do you think that this special ban is effectively implemented? (SINGLE: yes, no, don't know)

IV. PART FOUR: CONSERVATION & DEVELOPMENT

27. a) Do you benefit from a conservation and/or development program in your area? (SINGLE: yes, no, don't know)

27.b) If yes, how? (OPEN)____

____

28. Who/which organization leads this program? (OPEN)_____________________

____

29.a) Do you think that this program contributes to development in your community? (SINGLE: yes, no, don't know)

29.b) If yes, how? (ANY: education, alternative animal protein, direct cash-income, capacity-building, infrastructure development, other)

29.c) If other, what? (OPEN)____

30.a) Do you think that this program contributes to wildlife conservation in this area? (SINGLE: yes, no, don't know)

30.b) If yes, how? (ANY: reduced hunting, reduced logging, reduced mining, reduced use of other natural resources, better knowledge, positive attitudes towards wildlife, other)

30.c) If other, what? (OPEN)____

____

V. PART FIVE: EBOLA – KNOWLEDGE PRODUCTION

31.a) Have you heard of Ebola before? (SINGLE: yes, no) (→*if respondent answers no to this question, only ask about respondent's salary and local meat prices and stop part I of the interview afterwards*)

31.b) If yes, what causes it? (OPEN)____

32. When did you first hear about Ebola in Liberia? (NUM, month & year)_____

____

33.a) Do you know of people in THIS community or NEARBY communities that contracted Ebola? (SINGLE: yes, no)

33.b) If yes, when was this (NUM, month & year)___________________________

33.c) If yes, how many people do you know were infected with Ebola? (NUMBER: no. people infected)_______

33.c) If yes, how many of these died? (NUMBER: no. people dead)___________

34.a) Have you been informed about the risks of Ebola? (SINGLE: yes, no)

34.b) If yes, how? (ANY: radio, TV, posters, people from awareness campaigns, friends & colleagues, other)

34.c) If other, what? (OPEN)____

35.a) Is Ebola dangerous? (SINGLE: yes, no, don't know)

35.b) Why do you think that? (→*ask even if previous answer is "don't know"*) (OPEN)____

____

36.a) Where do you think Ebola originally came from? (SINGLE: African rainforest, other, don't know)

36.b) If other, specify (OPEN)____

____

____

37.a) Do you believe that people can contract Ebola from some or all species of bushmeat? (SINGLE: yes, no, don't know)

37.b) If yes, do you think that people can contract Ebola from preparing bushmeat, from eating bushmeat or from both? (SINGLE: preparing, eating, both, don't know)

37.c) If yes, which bushmeat species do you think can transmit Ebola? (OPEN)____

____

37.d) If yes, have you changed the way you prepare/cook bushmeat now after the Ebola crisis? (SINGLE: yes, no, don't know)

37.e) If no, from what else do you think can people contract Ebola? (OPEN)____

____

38.a) Do you know if people kill specific animal species because they are scared of getting Ebola from them? (SINGLE: yes, no, don't know)

38.b) If yes, which species? (OPEN)____

____

VI. PART SIX: EBOLA & BUSHMEAT CONSUMPTION

39.a) Did you eat bushmeat BEFORE the Ebola crisis? (SINGLE: yes, no, don't know)

39.b) If yes, how often did you eat bushmeat BEFORE the Ebola crisis? (SINGLE: with every meal, every day, every second day, twice a week, once a week, once a month, <once a month, don't know)

39.c) If yes, which animal species did you eat BEFORE the Ebola crisis? (OPEN)____

____

____

39.d) If yes, where did you get the bushmeat BEFORE the Ebola crisis? (ANY: forest, nearby market, friend, neighbour, large town, only through connections, other)

39.e) If other, what? (OPEN)____

40.a) What proportion of people in THIS community/NEARBY communities do you think preferred to eat bushmeat BEFORE the Ebola crisis? (SINGLE: everybody, half, >half, very few people, other)

40.b) If other, what? (OPEN)____

41. How many bodies of bushmeat did you purchase/consume in a typical week BEFORE the Ebola crisis? (NUM: # bodies)____________

FOLLOW-UP OF AFRICA WIDE GREAT APE POPULATION SURVEILLANCE / LIBERIA 2015

N°

42.a) Do you NOW eat less, more or the same amount of bushmeat as compared to before the Ebola crisis? (SINGLE: less, more, the same, don't know)

42.b) If answer = less, why do you eat less NOW during the Ebola crisis? (ANY: scared of getting Ebola, not easily available, too expensive, other)

42.c) If other, what? (OPEN)____________________________________

42.d) If answer = not easily available, or = too expensive, why do you think that? (ANY: less hunting, bushmeat market has moved "underground", closed markets, transport costs too high, roads are too bad, other)

42.e) If other, what? (OPEN)____________________________________

43. How often do you eat bushmeat NOW during the Ebola crisis? (SINGLE: with every meal, every day, every second day, twice a week, once a week, once a month, < once a month, never, don't know)

44. How many bodies of bushmeat do you purchase/consume in a typical week NOW during the Ebola crisis? (NUM: # bodies)____________________

45.a) What proportion of people in THIS community or NEARBY communities do you think prefer to eat bushmeat NOW during the Ebola crisis? (SINGLE: everybody, half, >half, very few people, other)

45.b) If other, what? (OPEN)____________________________________

46.a) Do you eat different meat NOW as compared to before the Ebola crisis? (SINGLE: yes, no, don't now)

46.b) If yes, which animal species do you eat NOW during the Ebola crisis? (OPEN)____

____

47.a) Where do you get bushmeat NOW during the Ebola crisis? (ANY: forest, nearby market, friend, neighbour, large town, only through connections, other)

47.b) If other, what? (OPEN)____________________________________

48. When last did you eat bushmeat? (SINGLE: today, yesterday, last week, last month, >2 months ago, >6 months ago, last year, several years ago)

VII. PART SEVEN: EBOLA & POTENTIAL VECTORS

49.a) Are bats resident in THIS community or NEARBY communities? (SINGLE: yes, no, don't know)

49.b) If yes, where do they sleep/rest? (ANY: house, tree, cave, don't know, other)

49.c) If other, where? (OPEN)___________________________________

50.a) Do people in THIS community or NEARBY communities eat bats? (SINGLE: yes, no, don't know)

50.b) If yes, what proportion of people in THIS community or NEARBY communities do you think may eat bats? (SINGLE: everybody, half, >half, very few people, other)

50.c) If other, what? (OPEN)____________________________________

51.a) If yes, do you think that the proportion of people who eat bats in THIS community or NEARBY communities changed due to the Ebola crisis? (SINGLE: yes, no, don't know)

51.b) If yes, is it more or less now than before the Ebola crisis? (SINGLE: more, less, don't know)

52.a) Have you noticed bats in fruit trees in this community/nearby communities? (SINGLE: yes, no, don't know)

52.b) If yes, how many? (SINGLE: very few, few, many, a lot, don't know)

52.c) If yes, did people frequently eat the fruits of the trees that had bats in them BEFORE the Ebola crisis? (SINGLE: yes, no, don't know)

52.d) If yes, do people still frequently eat the fruits of the trees that have bats in them NOW during the Ebola crisis? (SINGLE: yes, no, don't know)

53.a) Have you seen any dead chimpanzees that were NOT KILLED BY PEOPLE in the forest since the Ebola crisis? (SINGLE: yes, no, don't know)

53.b) If yes, how many? (NUM, no. of dead chimpanzees)__________

VI. PART SIX: EBOLA & EDUCATION

54.a) Is there a school in THIS community or NEARBY communities? (SINGLE: yes, no, don't know)

54.b) If yes, was school interrupted during the Ebola crisis? (SINGLE: yes, no, don't know)

54.c) If yes, for how long (NUM, no. of days/months*→circle correct unit!*) ______________**days/months**

VII. PART SEVEN: EBOLA & MIGRATION

55.a) Do you know of people in THIS community or NEARBY communities that fled/left their home because of Ebola? (SINGLE: yes, no)

55.b) If yes, how many? (NUM, no. of people)____________________

55.c) If yes, where did they move to? (SINGLE: another country, a remote village with few people, into the forest, another town, Monrovia, other)

55.d) If other, what? (OPEN)__________________________________

56.a) Do you know of people in OTHER AREAS in Liberia *(→not in this or nearby communities!)* that fled/left their home because of Ebola? (SINGLE: yes, no,. don't know)

56.b) If yes, how many? (NUM, no. of people)____________________

56.c) If yes, where did they move to? (ANY: another country, a remote area with few people, into the forest, another town, Monrovia, other)

56.d) If other, what? (OPEN)__________________________________

VIII. PART EIGHT: EBOLA & SECURITY

57.a) Do you think that the crime rate in THIS community or NEARBY communities has changed? (SINGLE: yes, no, don't know)

57.b) If yes, has crime increased or decreased? (SINGLE: increase, decrease, don't know)

57.c) If yes, was this due to Ebola? (SINGLE: yes, no, don't know)

57.d). If yes, why do you think is Ebola affecting crime rate? (OPEN)

____

57.e) If not due to Ebola, why did it change? (OPEN)______________

____

58.a) If crime = increased, what type of crime are you referring to? (ANY: theft, burglary, set fire, money laundering, fraud, assault, physical violence, rape, murder, drug crime, child abuse, domestic violence, other, don't know)

58.b) If other, what? (OPEN)__________________________________

IX. PART NINE: EBOLA & SOCIAL INTERACTIONS

59.a) Has Ebola affected how you socially interact with people? (SINGLE: yes, no, don't know)

59.b) If yes, how? (ANY: no hand-shakes, keep distance to others, no touching, staying indoors, no social gatherings, isolation, no sexual relationships other)

59.c) If other, what? (OPEN)____

60. What do you do to protect yourself from Ebola? (OPEN)____________________

61.a) Do you know of any Ebola survivors in THIS community or NEARBY communities? (SINGLE: yes, no)

61.b) If yes, how is their health now? (good, bad, same as before, don't know)

61.c) If yes, how did other people in the(se) community(ies) react upon their return from hospital? (SINGLE: welcoming, hostile, indifferent, other, don't know)

61.d) If other, what? (OPEN)____

61.e) If answer = welcoming or = hostile, why do you think they reacted like this? (OPEN)____

61.f) Do you think that they were fully integrated back into their community? (SINGLE: yes, no, don't know)

61.g) If no, why not?____

X: PART TEN: EBOLA – SUPPORT SYSTEM

62.a) Do you know of people in THIS community or NEARBY communities who died of a disease other than Ebola or during labour in the past 12 months because of a lack of medical treatment? (SINGLE: yes, no, don't know)

62.b) If yes, which disease(s) (*or if labour, indicate so*)? (OPEN)______________

62.c) If yes, do you think that this lack of medical treatment was related to the current Ebola crisis? (SINGLE: yes, no, don't know)

62.d) If yes, how (ANY: lack of medication because borders were closed, lack of hospital space, medical staff did not treat patient because of fear of getting Ebola, delays, lack of laboratory equipment, patient was scared of going to the hospital, other)

62.e) If other, what (OPEN)____

63a). When you get sick, who do you turn to/where do you go? (ANY: hospital, local clinic, church, traditional healer, village chief, elders, I stay at home where my family cares for me, other)

63.b) If other, where____

63.c) Has the place where you go/ the person you turn to when you get sick, changed because of this Ebola crisis? (SINGLE: yes, no, don't know)

63.d) If yes, where did you go to/ who did you turn to before this Ebola crisis? (ANY: hospital, local clinic, church, traditional healer, village chief, elders, I stay at home where my family cares for me, other)

64.a) Did you see people who wore protected clothes (*i.e. full-body overalls, face masks, goggles etc.*) in THIS community or in NEARBY communities? (SINGLE: yes, no)

64.b) If yes, what was their task? (ANY: medical staff, funeral teams, other)

64.c) If other, what? (OPEN)____

64.d) If yes, how did people react towards them? (ANY: happy, welcoming, scared, relieved, angry, aggressive, sad, other)

64.e) If other, how? (OPEN)____

XI: PART ELEVEN: EBOLA – GENERAL

65.a) How has Ebola affected your daily life? (ANY: Ebola had no effect, decrease in income, lack of goods other than food, food shortage, change in diet, change in occupation, displacement, death of relatives & friends, social discrimination, social isolation, ill health, constant fear, nightmares, crop loss, increased crime, other)

65.b) If other, what? (OPEN)____

65.c) If ≠ Ebola had no effect, how did you cope with this? (OPEN)

66.a) Do you think that the international community reacted appropriately to the current Ebola crisis in West Africa? (yes, no, don't know)

66.b) If answer = no, why not? (OPEN)_________________________________

67.a) Do you think that the Liberian government reacted appropriately to the current Ebola crisis in West Africa? (yes, no, don't know)

67.b) If answer = no, why not? (OPEN)_________________________________

68.a) Do you think that Ebola can come back again once this crisis is over? (SINGLE: yes, no, don't know)

68.b) If yes, how will you protect yourself/your family from Ebola in the future? (OPEN)____

69. What is your **monthly** salary/cash income? (NUM, amount → ***note currency!***)____

70. What is your occupation? (OPEN)______________________________________

71. Note local meat prices (**per whole body→*if another unit, translate into whole body, or note other unit!***) for the following animals (***note currency!***):

Chicken		Goat		Bushmeat: specify	
Beef		Sheep			
Fish		Pig			

72. Are you the head of your household? (SINGLE: yes, no)

73. End time. (NUM, time)__________________

74. Other comments: (OPEN)____

FOLLOW-UP OF AFRICA WIDE GREAT APE POPULATION SURVEILLANCE / LIBERIA 2014/2015

N° ________

This number has to MATCH the number of the questionnaire for the **SAME RESPONDENT** from the same village!!!!

Village name:____ **Start time:**_______________________________

GPS coordinates: Long____________________________ Lat_____________________________

1. **What is the size of your household** (household includes anybody who has been living in the family for more than 6 months)?

Male: age≤5__________, 5<age≤15__________, 16≤age≤ 64__________, age≥65__________

Female: age≤5__________, 5<age≤15__________, 16≤age≤64__________age≥65__________

(2.a) Only ask if respondent is NOT the household head - **what is the gender of the household head?** (M/F) __________)

(2.b) Only ask if respondent is NOT the household head - **what is the age of the household head?** (in years)__________)

3. **Has anyone recently died from an illness with high fever?** (SINGLE: yes, no)

3.a) Was anybody from your household/family infected by Ebola so far? (SINGLE: yes, no)

3.b) If yes, how many of your household members were infected? Give the details as follows:

Male: age ≤5__________, 5<age ≤15__________, age ≤15__________, 16≤ age ≤ 64_________age ≥65__________

Female: age ≤5__________, 5<age ≤15__________, age ≤15__________, 16≤ age ≤ 64_________age ≥65__________

3.c) How many of the above-reported people died? (NUM: no. of people who died)____________________

4.a) If yes, give the number of survivors as follows:

Male: age ≤5_________, 5<age ≤15_________, age ≤15__________, 16≤ age ≤ 64__________age ≥65__________

Female: age ≤5__________, 5<age ≤15__________, age ≤15__________, 16≤ age ≤ 64__________age ≥65__________

4.b) Has anyone from the above reported people received treatment? (SINGLE: yes, no).
4.c) If "yes", what kind of treatment? (SINGLE: local healer, local clinic, hospital, Ebola Emergency Unit, other)

4.d) If other, what? (OPEN)____
5. Do you own a farm? (SINGLE: yes, no)

6.i) If respondent is the household head ask: **What is your main occupation**?/If respondent is not the household head then ask: **What is the main occupation of the household head**? (SINGLE: farmer, professional (teacher, government worker, administration, health worker, clerical), skilled labourer (i.e. builder, thatcher, mason, etc),trader; 5= soldier; 6 =trader; 7= driver/mechanic; 8 =unskilled non-farm worker; 9 =domestic servant; 10= food-for-work; 11= cutting/sawing of planks from forest; 12= transport of planks from forest to the road side, 13= producing and selling local alcohol; 14=other, specify

6. What are the quantities and values of crops your household has harvested **during the past 12 months** (don't forget to include permanent crops like coffee, *Eucalyptus*, etc., as well as land reserved for grazing of livestock or fallow land)? (Please fill in the following table)

**Plot no.***	**Crops produced** (SINGLE: **code 1**)	**Area of production & unit** (e.g., ha)	**Total production** (report unit, e.g. kg)	**Sold, incl. barter** (same unit as column 4)	**Price/unit** (note currency!)	**Total amount of seeds used**** (report unit, e.g. kg)	**Total amount of fertilizer used** (report unit, e.g. kg)	**Price/ unit of fertilizer** (note currency!)	**Irrigation** Yes (Y)/No (N)

FOLLOW-UP OF AFRICA WIDE GREAT APE POPULATION SURVEILLANCE / LIBERIA 2014/2015

N°

***Code 1**: 1=maize; 2=beans; 3=cassava; 4=paddy; 5=sunflower; 6=onions; 7=millet; 8=Simsim; 9=groundnuts; 10=cashews; 11=tobacco; 12=sweet potato; 13=tomatoes 14=sugarcane; 15=coconut; 16=bananas; 17=mangoes; 18=papaya; 19=sorghum; 20=coffee; 21=tea; 22=cotton; 23=black wattle; 24=pine; 25=Cyprus sp.; 26=Eucalyptus sp.; 27=rice; 28=avocado; 29=pineapple; 30=guava; 31=pepper; 32=rubber; oil palm (Elaeis guineensis); 33=woodland; livestock grazing or left fallow; 31=other (specify), 32=other (**specify!**)*

***Same plot can be used more than once in different seasons or for more than one crop at the same time**

****Same unit as in column 4, or else clearly indicate unit here**

7. What are the quantities and values of inputs you used in crop production **during the past 12 months**? (Please fill in the following table)

**Inputs**	**Quantity**	**Unit**	**Price per unit** (note currency!)
Pesticides/herbicides			
Hired labor			
Hired machinery			
Payment for land rental			
Other (specify):			

8.a) Did you get credit/did you borrow money (that you will have to pay back) from any source in the past 12 months? (SINGLE: yes, no, don't know)

8.b) If yes, where did you get this credit from? (OPEN)______________________________

8.c) How much credit/money did you receive/borrow? (NUM: amount of money borrowed; **indicate currency**!)______________________________

9.a) Did you sell any crop residue in the past 12 months? (SINGLE: yes, no, don't know)

9.b) If yes, how much did you earn from the sale of crop residue? (NUM: amount of money; **indicate currency**!)______________________________

10.a) Is your household still working on your farm now? (SINGLE: yes, no)

10.b) If no, why? (SINGLE: got higher paying job, this time of the year is not harvest time, crops failed, need to care for ill relative, no farm land, fear of Ebola infection, other)

10.c) If other, why? (OPEN)______________________________

____

10.d) How do you compare your total production in the past 12 months to that in the previous year? (SINGLE: increased, decreased, about the same, don't know)

10.e) If ≠ don't know, why? (OPEN)______________________________

____

10.f) What do you expect about your next year harvest? (SINGLE: increase, decrease, about the same, don't know)

10.g) If ≠ don't know, why? (OPEN)______________________________

____

11.a) Did you have any farm land that was not cultivated in the past 12 months? (SINGLE: yes, no, don't know)

11.b) If yes, why? (ANY: lack of labour to hire, lack of farm animals (working force), lack of money to buy inputs, to leave the land fallow, illness of family member due to Ebola, illness of family member due to another disease, death of family member due to Ebola, death of family member due to another disease, fear of Ebola infection, other)

11.c) If other, what (OPEN)______________________________

12.a) What is the number of domestic animals your household has, and how many have you sold, bought, slaughtered or lost **during the past 12 months**? (Please fill in the following table)

**Livestock**	**Beginning number** (12 months ago)	**Sold** (alive or dead, and incl. barter)	**Slaughtered for own use** (or gift given)	**Lost** (theft, died, etc.)	**Bought or received as a gift**	**New-borns from own stock**	**Price/adult animal** (average) Indicate currency!
Cattle							
Goats							
Sheep							
Pig							
Donkey							
Ducks							
Chicken							
Guinea pig							
Rabbit							
Turkey							

N°

Guinea fowl							
Other (specify)							

12.b) How do you compare your livestock ownership in the past 12 months to that in the year before? (SINGLE: increase, decrease, about the same, don't know)

12.c) If increase or decrease, what do you think was the reason for this change? (OPEN)____

____

13. What are the quantities and values of animal products and services that you have produced **during the past 12 months**? (Please fill in the following table)

**Product/service**	**Total Production**	**Unit**	**Own use (incl. gifts)**	**Sold (incl. barter)**	**Price per unit** (note currency!)
Milk					
Butter					
Cheese					
Eggs					
Hides and skin					
Wool					
Manure					
Honey					
Curdled milk					
Soap					
Other (specify):					

14.a) Did the household receive any other income (e.g. transferred money (remittance) from friends/relatives/others, gifts, food aid/other aid, **IMPORTANT: don't include credit that the respondent received in this table!**) **during the past 12 months**? (SINGLE: yes, no)

14.b) If yes, give the details in the following table:

**Type of income received** (SINGLE: **code 2**)	Amount received **during the past 12 months** (if in kind, give type of payment, amount, unit, and monetary value)				
	**Amount received in cash** (note currency!)	**Type of in-kind payment**	**Amount in-kind** (use appropriate unit!)	**Price of in kind payment** (note currency!)	How has Ebola influenced how much income you received over the past 12 months? (SINGLE: increased, decreased, about the same, don't know)***

*****Indicate answer for each row that includes data!***

***Code 2:** 1=remittance (transferred money), 2=gift, 3=inheritance, 4=foreign aid, 5=donation, 6=other (**specify!**)*

N°

FOLLOW-UP OF AFRICA WIDE GREAT APE POPULATION SURVEILLANCE / LIBERIA 2014/2015

15. Has the household faced any major income shortfalls or unexpectedly large expenditures **during the past 12 months**? (Please fill in the following table)

**Description of shortfall**	**Severity** (SINGLE: **code 3**)	**Coping strategies** (ANY: **code 4**)[1]					
		**1st**	**2nd**	**3rd**	**4th**	**5th**	
Serious crop failure							
Serious illness in family (person unable to work for more than one month **during past 12 months**, due to illness, or to taking care of ill person, or high medical costs)							
Death of productive age-group adult							
Land loss (expropriation, etc.) – *not the same as "not being able to work on farm"!*							
Major livestock loss (theft, drought, etc.)							
Other major asset loss (fire, theft, flood, etc.)							
Lost wage employment							
We couldn't work on the farm due to Ebola							If yes, how many days could you **NOT** work on the farm? (NUM: days)________
Wedding or funeral or other costly social event							
Payment for sale of household products arrive later than expected							
Other (specify):							

[1]Please probe for strategies one by one. Read out all strategies to the respondent and ask him if it applies to his household or not, only list in the table the strategies that do apply and specify if "other" (e.g. 2, 10, 22 (played the lottery ☺)). Also, rank each strategy according to its importance, i.e. 1st to 5th, from most important to least important and fill into appropriate columns.

***Code 3**: 1=no crisis, 2=yes, moderate crisis, 3=yes, severe crisis*

***Code 4**: 1=harvest more forest products, 2=harvest more wild products not in the forest, 3=go hunting, 4=harvest more agricultural products, 5=spend cash savings, 6=sell assets (land, livestock, etc.), 7=do extra casual labour work, 8=assistance from friends and relatives, 9=assistance from NGO/community org./religious org. or similar, 10=get loan from money lender/credit association/bank etc., 11=tried to reduce household spending, 12=spent savings/retirement money, 13=reduced number of meals eaten per day, 14=borrowed against future earnings, 15=sold food that would otherwise be used for household consumption and/or seed for the next season, 16=rented out land, 17=started new business, 18=changed to different type of livestock, 19=harvested premature crops; 20=changed cropping patterns or types of crops planted, 21=did nothing in particular, 22=other (**specify!**)*

16.a) Did you or any other member of your household seek additional employment for payment in cash or in kind **during the past 12 months**? (SINGLE: yes, no)

16.b) If yes, give the details in the following table:

**Type of employment** (SINGLE: **code 5**)[2]	Total amount earned **in the last 12 months** from off- own-farm employment (if in kind, give type, amount, unit, and price)				
	**Amount received in cash** (note currency!)	If in kind, the **type of in-kind payment**	**Amount in-kind** (use appropriate unit!)	**Price of in kind payment** (note currency!)	How has Ebola influenced how much income you received over the past 12 months? (SINGLE: increased, decreased, about the same, don't know)[3]

***Code 5:** 1=farm worker (on somebody else's farm) for pay, 2=professional (e.g. teacher, government worker, administration, health worker, clerical, etc.), 3=skilled labourer (e.g. builder, thatcher, mason, etc), 4=trader, 5=soldier, 6=driver/mechanic, 7=unskilled non-farm worker, 8=domestic servant, 9=food-for-work, 10=cutting/sawing of planks from forest, 11=transport of planks from forest to the road side, 12=other (**specify!**)*

[2]Please probe for strategies one by one. Read out all strategies to the respondent and ask him if it applies to his household or not, only list the strategies that do apply (e.g. 2, 5, 12 (prostitution))

[3]Indicate answer for each row that includes data below!

N°

17.a) Is the diversity of food items you eat **NOW DURING** the Ebola outbreak the same as **BEFORE** the Ebola outbreak? (SINGLE: yes, no, don't know)
17.b) If no, how has it changed? (SINGLE: increased, decreased, don't know)
18.a) Did you/your household face food shortages in the past 12 months? (SINGLE: yes, no)
18.b) If yes, what was the reason for this food shortage? (ANY: 1=usually we face shortage in this season, 2=we couldn't plant crops that lasted until the end of this season due to Ebola; 3= that lasted until the end of this season due to too little rain, 4=we couldn't work to earn income to spend on food due to Ebola, 5=there was a shortage of food items in the market, 6=the price for food items was too high, 7=other)

18.c) If other, specify (OPEN)____

____

18.d) If yes (i.e. there was a shortage), how did you cope with this shortage (**code 6**)? Please rank the coping strategies! (**Hint: first underline the respondent's answers and then read the answers to the respondent again and ask how importance each is for him/her!**)

1st	2nd	3rd	4th	5th

Code 6: *1=borrowing, 2=reduce number of meals and amount eaten, 3=eat less preferred food, 4=use savings to purchase food, 5=harvest more wild food from the forest, 6=sell assets (land, livestock, etc.), 7=do extra casual labour work, 8=assistance from friends and relatives, 9=assistance from NGO/community org./religious org. or similar, 10=get loan from money lender/credit association/bank etc., 11=borrowed against future earnings, 12=rented out land, 13=harvested premature crops, 14=changed cropping patterns or types of crops planted, 15=sent out part of my children to relatives, 16=restrict adult food consumption to feed children, 17=selling out house and/or furniture, 18=did nothing in particular, 19=other* ***(specify!)***

19.a) Did you eat at least one cooked meal every day **BEFORE** the Ebola crisis? (SINGLE: yes, no)

19.b) Do you eat at least one cooked meal every day **NOW DURING** Ebola crisis? (SINGLE: yes, no)

20.a) How many times a day (on average) did you eat **BEFORE** the Ebola crisis? (NUM: no. of times/day)________________

20.b) How many times a day (on average) do you eat **NOW DURING** the Ebola crisis? (NUM: no. of times/day)________________

21. Which of the following food items did you eat **in past 7 days**? (Please fill in the following table)

**Did you eat…….in the past 7 days?** (**underline** each of the appropriate food items)	**If other, specify here**	**On how many days did you eat …… in the past 7 days?** (use numbers of appropriate food items in each row and indicate number of days the item was eaten in the past 7 days, e.g. 1=2; 5=5)	**From where did you get the food item?** (ANY: **code 7**) (use numbers of appropriate food items in each row and indicate correct code for each, e.g. 1=2; 5=7 (found))
Staples: cassava[1], sweet potato[2], corn[3], rice[4], plantain[5], wheat (e.g. in bread)[6], other[7]			
Vegetable/greens/salad: tomato[1], potato greens[2], cassava leaves[3], cabbage[4], pumpkin[5], bitter root[6], bitter balls[7], other[8]			
Grain legumes & nuts: red kidney beans[2], other beans[3], lentils[4], peanuts & peanut butter[5], other nuts[6], other[7]			
Fruit: banana[1], orange[2], lemon[3], guava[4], mango[5], avocado[6], pineapple[7], coconut[8], other[9]			
Meat: beef[1], chicken[2], pork[3], duck[4], goat[5], sheep[6], bush meat[7], turtle[8], other[9]	If bushmeat, specify species:________________________ ____________________________		
Fish, shellfish & other marine and freshwater animals: fresh-water fish[1], salt-water fish[2], crabs[3], crayfish[4], tortoise[5], other[6]			
Eggs: chicken eggs[1], duck eggs[2], wild bird eggs[3], tortoise eggs[4], crocodile eggs[5], other[6]			
Sugar[1]			
Milk: cow milk[1], goat milk[2], sheep milk[3], other[4]			
Oil: palm oil[1], white vegetable oil[2], other[3]			

Code 7: *1=own production, 2=purchased, 3=food for work, 4=food aid, 5=gift, 6=borrowed, 7=other* ***(specify!)***

22.a) If you compare a typical meal **BEFORE** and **NOW DURING** the Ebola crisis, is there any difference? (SINGLE: yes, no, don't know)
If yes, answer the following 2 questions:
22.b) On a typical day **BEFORE** the Ebola crisis, what would a typical meal consist of? (OPEN)____

____

22.c) On a typical day **NOW DURING** the Ebola crisis, what would a typical meal consist of? (OPEN)____

____

23. How much do you agree with the following statements (**mark appropriate answer with an "X"**)?

Statement:	Strongly agree	Agree	Neutral	Disagree	Strongly disagree	Has Ebola changed this level of trust? (**code 8**)
Most of the people in my community are trustworthy						
Most of the people in the neighboring community are trustworthy						
Most of the people in my community are willing to help if needed						
Most of the people in my tribe are trustworthy						
Most people of the people who belong to the same religion as I are trustworthy (**if no religion leave blank!**)						
The government of Liberia is trustworthy						
The head of my community is trustworthy						
International aid is trustworthy						
Medical workers are trustworthy						

Code 8: *1=increased, 2=decreased, 3=about the same, don't know)*

24. Rank (give **numbers from high to low** - **from most important to least important**) the following according to the importance in case you need to get help from somebody in the case of emergency: family member_____, neighbour_____, friend_____, colleague (from work)_____, church member_____, police (other government)______, other (**specify!**)___________________________

25.a) In which of the following collective/group actions are you and/or any member of your household engaged in? (**Read out all possible answers to the respondent & underline the ones that are correct!**) (ANY: road construction/maintenance, school construction, digging drinking water pits, wildlife management, forest management, ecotourism, church construction/maintenance, community services (e.g. woman representative, youth representative, etc., other)

25.b) If other, specify (OPEN)____

26.a) Are you a member of any of the following organizations/institutions in your community? (**Read out all possible answers to the respondent & underline the ones that are correct!**) (ANY: local government administration, other government office, church, social club, savings club, NGO, other)

26.b) If other, specify (OPEN)____

26.c) If yes, did Ebola affect your involvement in the any of these institutions/organizations? (SINGLE: yes, no, don't know)

26.d) If ≠ don't know, how? (OPEN)____

____

27.a) **Ask this only if the respondent IS part of an organization/institution:** Do you hold any official position? (SINGLE: yes, no)

27.b) If yes, what is the name of this organization? (OPEN)____

End time:________________________________
